# Exploring the Risk of Suicide in Real Time on Spanish Twitter: Observational Study

**DOI:** 10.2196/31800

**Published:** 2022-05-17

**Authors:** Claudia García-Martínez, Bárbara Oliván-Blázquez, Javier Fabra, Ana Belén Martínez-Martínez, María Cruz Pérez-Yus, Yolanda López-Del-Hoyo

**Affiliations:** 1 Department of Psychology and Sociology University of Zaragoza Zaragoza Spain; 2 Department of Psychology and Sociology University of Zaragoza Institute for Health Research Aragón Zaragoza Spain; 3 Department of Computer Science and Systems Engineering Aragón Institute of Engineering Research University of Zaragoza Zaragoza Spain; 4 Department of Nursing and Physiatry Institute for Health Research Aragón University of Zaragoza Zaragoza Spain

**Keywords:** suicide, prevention, social media, Twitter, emotional analysis, eHealth, big data, content analysis, emotional content, risk factors, mental health, public health, suicide prevention

## Abstract

**Background:**

Social media is now a common context wherein people express their feelings in real time. These platforms are increasingly showing their potential to detect the mental health status of the population. Suicide prevention is a global health priority and efforts toward early detection are starting to develop, although there is a need for more robust research.

**Objective:**

We aimed to explore the emotional content of Twitter posts in Spanish and their relationships with severity of the risk of suicide at the time of writing the tweet.

**Methods:**

Tweets containing a specific lexicon relating to suicide were filtered through Twitter's public application programming interface. Expert psychologists were trained to independently evaluate these tweets. Each tweet was evaluated by 3 experts. Tweets were filtered by experts according to their relevance to the risk of suicide. In the tweets, the experts evaluated: (1) the severity of the general risk of suicide and the risk of suicide at the time of writing the tweet (2) the emotional valence and intensity of 5 basic emotions; (3) relevant personality traits; and (4) other relevant risk variables such as helplessness, desire to escape, perceived social support, and intensity of suicidal ideation. Correlation and multivariate analyses were performed.

**Results:**

Of 2509 tweets, 8.61% (n=216) were considered to indicate suicidality by most experts. Severity of the risk of suicide at the time was correlated with sadness (*ρ*=0.266; *P*<.001), joy (*ρ*=–0.234; *P*=.001), general risk (*ρ*=0.908; *P*<.001), and intensity of suicidal ideation (*ρ*=0.766; *P*<.001). The severity of risk at the time of the tweet was significantly higher in people who expressed feelings of defeat and rejection (*P*=.003), a desire to escape (*P*<.001), a lack of social support (*P*=.03), helplessness (*P*=.001), and daily recurrent thoughts (*P*=.007). In the multivariate analysis, the intensity of suicide ideation was a predictor for the severity of suicidal risk at the time (*β*=0.311; *P*=.001), as well as being a predictor for fear (*β*=–0.009; *P*=.01) and emotional valence (*β*=0.007; *P*=.009). The model explained 75% of the variance.

**Conclusions:**

These findings suggest that it is possible to identify emotional content and other risk factors in suicidal tweets with a Spanish sample. Emotional analysis and, in particular, the detection of emotional variations may be key for real-time suicide prevention through social media.

## Introduction

As the cause of more than 800,000 deaths every year, suicide has become a global public health priority. It is the second leading cause of death in young people aged 15 to 29 years, and for every death, it is estimated that there are 20 other suicide attempts [1]. In Spain, suicide has been the main cause of unnatural death since 2012 [2].

According to the World Health Organization [3], suicidal behavior refers to a range of behaviors that includes thinking about suicide (or ideation), planning to commit suicide, attempting suicide, and suicide itself.

Until a few decades ago, research efforts have been focused on curbing suicide deaths by trying to predict their occurrence. This predictive approach consisted of semistructured risk assessment using lists of risk factors and sometimes included suicide risk questionnaires or scales to express risk as low, moderate, or high [4,5]. Because suicide deaths are statistically a rare event, it has been difficult to develop sensitive tools with sufficient predictive value [6]. Recent reviews [5,7-10] of these models advocate a shift from models based on suicide prediction to those that emphasize assessment and management of the risk of suicide by identifying variables related to suicide behavior and stratifying risk in terms of severity and temporality.

Suicidal behavior has been consistently found to be associated with emotional states such as depression and hopelessness [11,12]. Bryan and Rudd [9] collected different variables that have been empirically demonstrated to be essential for risk assessment: predisposition to suicidal behavior (ie, psychiatric diagnoses, previous suicidal behavior), identifiable precipitants or stressors (ie, significant loss, relationship instability), a patient’s symptomatic presentation (eg, anhedonia, low self-esteem, sadness, dyssomnia, fatigue), presence of hopelessness, nature of suicidal thinking (eg, ideation, suicidal plan, lethality of means, explicit suicidal intent), impulsivity and self-control, and protective factors (eg, social support, life satisfaction). Emotional dysregulation seems to be also an important predictor of suicidal outcomes [13,14].

To improve accuracy in risk evaluations, ecological momentary assessment has been used to study suicidal behavior [15], which involves repeated sampling of people's behavior in real time in their natural environments, now typically collected via smartphones. This approach attempts to minimize recall bias and maximize ecological validity. Recent research using mobile phone–based momentary ecological assessments showed that suicidal ideation varied over short periods of time [16], indicating that real-time assessments and ecological validity could be a crucial approach for suicide prevention.

With more than 3.8 billion users around the globe [17], social media has transformed the world. People express their thoughts and emotions through social media [18]. These new forms of social interaction have been linked to suicidal behavior, nevertheless, recent studies [19] have highlighted the potential for social media to offer assistance in suicide prevention.

Twitter currently has 340 million users [20] who, in microblogging format, communicate what they are thinking or doing at a particular moment publicly with a limited number of characters. People express suicidal tendencies on Twitter [21], and although there is a support mechanism among users, this system is not automatic or in real time.

According to Christensen [22], web-based interventions for suicide prevention have focused mainly on three directions: (1) web-based screening for suicidality, (2) web-based therapeutic interventions for suicide prevention, and (3) real-time identification of individuals at risk, either by people or by computer language processing systems.

The use of social media in the real-time detection of mental health has already been proven. Specifically, Twitter has been proven useful in predicting depression [23-25], postpartum depression [24], and even posttraumatic stress disorder [26,27]. Machine learning algorithms have been used to assess the risk of suicide and identify suicidal individuals. Automatic machine learning classification systems that are able to effectively differentiate people who are at risk of suicide from those who are not [28-30] and identify temporal patterns in posts before suicide [31] have been developed. Reviews on the subject [22,32] yield similar results: social media is an empirically tested tool for suicide detection, but further validation is needed.

Recent studies have incorporated human coders in order to create language classification systems or validate machine learning results from natural language processing systems [33]. Nevertheless, the need to incorporate mental health experts in suicide assessments for the improvement of accuracy has been noted [34]. Furthermore, the detection of the risk of suicide for social media users located in Spain has yet to be explored.

Our objective was to analyze the risk of suicide among Twitter users who post in Spanish, by assessing the emotional content of their posts and other variables that have been identified as being related to suicidal behavior, such as perception of defeat, helplessness, and social support.

## Methods

### Design

**Figure 1:**
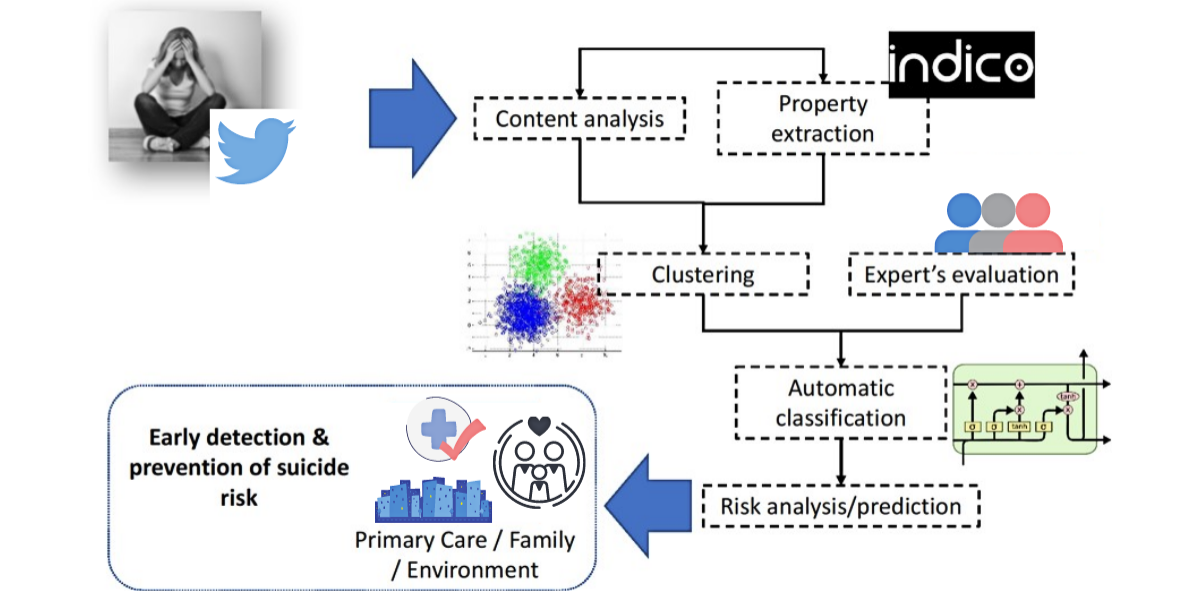
Methodology for the early detection and prevention of the risk of suicide on Twitter.

We conducted a cross-sectional exploratory study. To collect and analyze tweets, we used a framework based on computer technologies that we had developed previously [35]. This is a full framework (Figure 1) that has been engineered and implemented using various technologies and has been structured around a multidisciplinary team of professionals from health sciences and professionals with specialization in information technology. We focused on the Expert’s evaluation stage. The first step was to obtain the Twitter entries from keywords related to suicide. To identify potentially emotional tweets, a large vocabulary of emotional terms was compiled from different sources, including the Spanish adaptation of Affective Norms for English Words [36], which provides a set of emotional normative scales for a set of words, and the Spanish dictionary of the Linguistic Inquiry and Word Count [37], which is an analysis software that calculates the degree to which people use different categories of words across a wide spectrum of texts. The use of Linguistic Inquiry and Word Count software to assess positive and negative emotions has been validated [38,39].

Adding properties to the text contained in the tweet facilitates and improves the identification and classification of groups at risk of suicide. Therefore, a series of properties associated with the text were obtained and added. The properties were based both on external natural language processing systems and platforms, and on internal algorithms that obtained the information through a text evaluation platform, which is completed by selected reviewers. The emotional vocabulary was organized by combining the hierarchy of emotions [40] and the tree of emotions [41]. Each emotional word was classified into 6 categories of primary emotions (love, joy, surprise, anger, sadness, and fear) and 25 subgroups of secondary emotions using affective and emotional text processing software (Indico, version 2020) that provides a toolkit of application programming interfaces (APIs). We used the following APIs for text-based analysis: Sentiment Analysis, Text Tags, Language Detection, Emotions, Personality and Personas. These APIs do not support the use of emoticons, and they ignore the appearance of these elements during text processing. A low or moderate use of emojis was observed in the content captured from Twitter that were relevant to the study. However, given that the use of emojis is ubiquitous, this fact must be taken into account when extending this work. The study of the criteria for matching between emojis and suicide or suicidal tendency is an aspect that we propose in future work in the short term.

To cluster data groups with similar characteristics—in our case, we wanted to identify groups at risk of suicide—we selected *k*-means clustering [42]. This method is based on partitioning data into *k* well-defined groups. To select the *k* value, a cross-validation method was used [43].

The clusters were analyzed in order to understand the characteristics of each one. This step needed to be carried out by a group of reviewers who were experts in the subject in order to understand the quality of the groups. The objective of this analysis was to validate that the clusters corresponded to groups at risk of suicide. At this stage, human coding was used to determine the degree of relationships of the classified tweets, based on the judgment of researchers from the fields of mental health and medicine who specialized in suicide prevention and had training in detecting the risk of suicide.

An automatic classifier with clusters and tweets as input was created. This classifier was capable of receiving new tweets and classifying them into one of the groups in order to determine whether there was a risk of suicide or not. We used a long short-term memory neural network.

Tweets were selected using Twitter’s API. Because most Twitter accounts were not geolocated, we selected posts written in Spanish. We checked this using both the Twitter information obtained through the API and by using a Language Detection API (Indico). We excluded posts in Latin-Spanish language because the cultural context of the tweet could be unknown. These tweets were collected between November 2019 and February 2020. The study included 25 psychologist evaluators with clinical or research experience who were trained to assess the tweets for the variables of the study. Each tweet was randomly assigned to 3 different evaluators. Each expert evaluated a tweet independently, without knowing to which other evaluators the tweet had been assigned. Assessments were carried out using a smartphone-based software. The tweets were evaluated between the months of March and April 2020.

### Study Variables

#### Primary

Only posts from the data set that were considered to be relevant by most experts were subsequently analyzed. Tweets were considered relevant if the content of the text was related to a potential risk of suicide of the author of the tweet. Tweets in which—(1) the text did not correspond to content related to a possible risk of suicide or associated emotional states; (2) the text was written in a language other than Spanish or the text was written in Latin Spanish; (3) the text of the tweet was ambiguous (ie, the context was unknown); or (4) the text of the tweet was not suicide-relevant for any other reason were and discarded.

#### Secondary

##### Outcome Variable

The outcome variable was the severity of the risk of suicide at the moment of writing the tweet. We used a scale based on a suicide risk continuum with 5 levels [9]: (0) nonexistent risk, (1) mildly suicidal, (2) moderately frequent, (3) severely frequent, and (4) extremely frequent with intense and enduring suicidal ideation, specific plans, clear subjective and objective intent, impaired self-control, severe dysphoria, many risk factors, and no protective factors (ie, extreme risk).

##### Valence

Valence represented if the emotional valence of the tweet was positive or negative. If the value was greater than 50, it was considered that the text expressed a positive or pleasant feeling. If it was less than 50, it was considered to have a negative valence, that is, it expressed a negative, aversive, or unpleasant feeling. This was assessed on a scale from 0 and 100, using 2 decimal places.

##### Emotional Content

The level of anger, joy, fear, sadness, and surprise expressed in each tweet was evaluated on a scale from 0 and 100 (with 2 decimal places), with values closest to 100 being the highest level of the emotion.

##### Relevant Personality Traits

Traits—extroversion, if the author of the text showed extroversion(ie, is a person who is focused and interested in the outside world); sensory, if the author has a sensory tendency when processing information (ie, is a person who pays attention to details and prefers to work with concrete facts than with speculation or possibilities); rational, if the person has a tendency to make decisions based on logic, using an analytical and objective approach(ie, is a person who supports their decisions with impersonal analysis rather than with personal values); and judgment, if the author of the post has a preference for a planned (stable and organized, rather than spontaneous and flexible) life—were evaluated on a scale between 0 and 100 (with 2 decimal places), with values closest to 100 being the closest to that trait. These personality traits were collected from Myers-Briggs Type Indicators [44].

##### Other Variables

Other relevant variables on suicide risk assessments were also collected in the event that the information available was sufficient to assess them (or left blank if the parameter was not identifiable in the content of the tweet): (1) feelings of defeat, rejection, or both, if it was possible to identify a stressful event that generated feelings of defeat, rejection, or both feelings in the text of the author; (2) desire to escape from the situation, or if desire or will to run from a situation can be identified, evaluated using a dichotomous scale (yes or no); (3) social support or possibility of perceived help was evaluated dichotomously (yes or no); (4) feelings of helplessness or lack of coping resources were evaluated dichotomously (yes or no); (5) the general risk of suicide for the author of the tweet was evaluated on a scale between 0 and 4, with 0 being no risk and 4 being an extreme risk; (6) daily recurring thoughts of suicide was evaluated dichotomously (yes or no), and the intensity of autolytic thoughts was assessed on a scale from 0 to 10, with 0 being not intense and 10 being very intense; (7) content related to the tweet author’s sleep, insomnia, or hypersomnia was evaluated dichotomously (yes or no).

### Statistical Analysis

Statistical analyses were conducted using SPSS software (version 22; IBM Corp). First, the sample distribution was analyzed. Kolmogorov-Smirnov values <0.05 were obtained for all variables; thus, nonparametric statistics were used. For quantitative variables, median and interquartile range were calculated, and for qualitative variables, frequency and percentages were calculated. The dependent variable (the severity of the risk of suicide at the present time) was analyzed as a continuous scale with a minimum of 0 and a maximum of 4. Spearman correlations between the severity of the risk of suicide at the time and the variables were calculated. Severity of the risk of suicide at the time was compared between qualitative variables using the Mann-Whitney *U* test (when there were 2 different groups) or the Kruskal-Wallis test (when there were more than 2 groups). A multivariate model was developed for severity of the suicidal risk at the time of tweeting. The independent variable was added into the regression model [45], and a final model was obtained. Linear regression was used since the residuals of the model had a finite mean, constant variance, and normal distribution (above all, because the sample size was very high; with the central limit theorem, any distribution with constant mean and variance, if it has a large enough sample size, has a normally distributed mean). However, bootstrapping analysis with 2000 samples was also conducted. The mean value of the 2 or 3 evaluators was used for continuous and qualitative variables, and the coinciding value between 3 evaluators, or 2 of 3 evaluators, was used. The interrater reliability was calculated using Fleiss *κ*. *P* values <.05 were considered to be significant.

### Ethical Issues

All procedures contributing to this work complied with the ethical standards of the Clinical Research Ethics Committee of Aragón (Department of Health, Government of Aragón, Spain) and with the Helsinki Declaration of 1975, as revised in 2013 [46]. The study protocol was approved by the Clinical Research Ethics Committee of Aragón, Spain (17/0127, with the number PI21/164).

## Results

A total of 2509 tweets were obtained, of which 2018 were deemed not relevant by 3 evaluators, and 275 were deemed not relevant by 2 of 3 evaluators. There were 216 tweets that were found to be relevant by most evaluators, with 68 tweets considered to be relevant by all evaluators, exhibiting moderate reliability (Fleiss *κ*=0.41).

Tweets mainly conveyed sadness and defeat, with there being no desire to escape, no support, and no feelings of helplessness (Table 1). The median overall risk of suicide was 1.50 (IQR 1.00) on a scale from 0 to 4. The median severity of risk was 1.00 (IQR 1.16); 96.9% (186/192) of tweets did not indicate the presence of daily recurring thoughts of suicide, and the median intensity of suicidal thoughts was 4.50 (IQR 3.00) on a scale from 0 to 10.

**Table 1:** Description of the tweets deemed relevant.

Variables			Value
Valence^a^, median (IQR)			21.58 (24.25)
**Emotional content^a^ (n=216), median (IQR)**			
	Anger	24.00 (34.00)	
	Joy	0.00 (1.50)	
	Fear	17.25 (32.37)	
	Sadness	51.41 (39.12)	
	Surprise	0.50 (5.50)	
**Relevant personality traits^a^ (n=216), median (IQR)**			
	Extroversion	28.00 (34.29)	
	Sensory	25.16 (29.25)	
	Rational	19.50 (27.37)	
	Judgement	19.00 (32.00)	
**Feelings of defeat or rejection (n=98), n (%)**			
	Defeat	61 (62.2)	
	Rejection	16 (16.3)	
	Both	21 (21.4)	
**Desire to escape (n=161), n (%)**			
	Yes	25 (15.5)	
	No	136 (84.5)	
**Social support or possibility of perceived help (n=196), n (%)**			
	Yes	4 (2.0)	
	No	192 (98.0)	
**Feelings of helplessness (n=152), n (%)**			
	Yes	57 (37.5)	
	No	95 (62.5)	
**Suicide risk variables^b^ (n=216), median (IQR)**			
	General risk	1.50 (1.00)	
	Severity suicidal risk at present moment (real-time risk)	1.00 (1.16)	
**Daily recurrent thoughts of suicide (n=192), n (%)**			
	Yes	6 (3.1)	
	No	186 (96.9)	
Intensity of autolytic thoughts^c^, median (IQR)			4.50 (3.00)
**Content related to insomnia or hypersomnia (n=210), n (%)**			
	Yes	4 (1.9)	
	No	206 (98.1)	

^a^These variables were evaluated on a scale from 0 to 100.

^b^These variables were evaluated on a scale from 0 to 4.

^c^This variable was evaluated on a scale from 0 to 10.

There were direct correlations between severity of the risk of suicide at the time of generating the tweet and sadness, general risk, and intensity of suicide thoughts, as well as inverse correlations with extroversion, rational trait, and joy (Table 2).

The severity of risk of suicide at the time of generating the tweet was higher in people who expressed feelings of defeat, rejection, desire to escape, feelings of helplessness, lack of social support, and daily recurrent thoughts (Table 3).

The linear regression model (*R*^2^=0.750; adjusted *R*^2^=0.710) showed that the intensity of autolytic thoughts, fear, and valence were predictors of the severity of the risk of suicide at the time (Table 4). Both the intensity of autolytic thoughts and valence had positive coefficients, and fear had a negative coefficient. This indicated that when intensity was higher, valence was more positive, and when fear was lower, the severity of suicidal risk was higher. The model explained 75% of the variance.

**Table 2:** Spearman correlations between variables and severity of the risk of suicide at the time of writing the tweet.

Variables			*ρ*		*P* value
Valence			–0.069		.31
**Emotional content**					
	Anger	–0.013		.85	
	Joy	–0.234		.001	
	Fear	–0.097		.16	
	Sadness	0.266		<.001	
	Surprise	–0.075		.27	
**Relevant personality traits**					
	Extroversion	–0.22		.001	
	Sensory	–0.115		.09	
	Rational	–0.244		<.001	
	Judgement	–0.128		.06	
**Suicide risk variables**					
	General risk	0.908		<.001	
	Intensity of autolytic thoughts	0.766		<.001	

**Table 3:** Comparison of severity of the risk of suicide at the time of writing the tweet between qualitative variables.

Variables		Severity of the risk of suicide at the moment (real-time risk), median (IQR)	*P* value
**Feelings of defeat or rejection**			
	Defeat	1.33 (1.42)	.003
	Rejection	0.33 (1.25)	
	Both	1.66 (1)	
**Desire to escape**			
	Yes	1 (0)	<.001
	No	1 (1.17)	
**Social support or possibility of perceived help**			
	Yes	0.33 (0.66)	.03
	No	1 (1.16)	
**Feelings of helplessness**			
	Yes	1.5 (1)	.001
	No	1 (1.17)	
**Daily recurrent thoughts of suicide**			
	Yes	2.16 (1.30)	.007
	No	1 (1)	
**Content related to insomnia or hypersomnia**			
	Yes	0.75 (0.63)	.22
	No	1 (1.16)	

**Table 4:** Linear regression model coefficients indicating the relationship to severity of the risk of suicide at the time of writing the tweet.

Variables	Coefficient (95% CI)	*P* value
Constant	0.110 (–0.169, 0.412)	.45
Intensity of autolytic thoughts	0.311 (0.250, 0.370)	.001
Fear	–0.009 (–0.015, –0.005)	.01
Valence	0.007 (0.002, 0.013)	.009

## Discussion

Suicide prevention is a crucial field that needs to be developed to stop preventable deaths worldwide. The findings of our study reveal that social media can be used to help to identify individuals at risk. These findings suggest that it is possible to identify suicidal behavior through Spanish tweets, and it is possible to identify these posts, not only by using a suicide-related lexicon but also, by filtering tweets based on their emotional content. Tweets that show sadness, defeat, and perceived lack of social support suggest that there is a risk of suicide. These variables have been commonly associated with symptoms of depression and hopelessness [12,47].

One of the main challenges in suicide prevention is identifying not only the people at risk of experiencing suicidal behavior at some point in their lives but those who are at risk at a particular moment, in our case, while they are writing the tweet.

Suicidal ideation and its risk factors can fluctuate over short periods of time [16], which demonstrates the importance of differentiating general suicide risk (or suicide status, such as in individuals with long-term risk factors) from real-time suicide risk. In this exploratory study, we emphasized assessment of suicide risk at the moment of writing the post. Our results provide some clues about the phenomenon of suicidal behavior.

In our study, although the *variable desire to escape from the situation* was not identifiable in most posts showing potential risk, it was related to an increase in the severity of the risk at the time of writing the tweet in posts that expressed a desire to escape.This outcome could suggest that the variable of the desire to run from a suffering situation may be only relevant in situations with an increased risk of suicide at that time. We found the same pattern for feelings of helplessness at the time, which was only identifiable in high-risk tweets. These results are consistent with recent conceptualizations of acute suicidal behavior that suggest that this feeling of entrapment—“in which the escape from an unbearable life situation is perceived as both urgent and impossible” [48]—is linked with imminent suicidal behavior. Nevertheless, further research is needed.

Individuals exhibiting high levels of negative urgency and emotion reactivity might be more likely to develop suicidal ideation and resort to self-harm while experiencing negative affective states [14,49]. In our sample, tweets with higher risk (at the time of writing the tweets) were identified by higher sadness, higher general risk, and higher intensity of suicidal thoughts on a daily basis. In addition, they similarly showed feelings of defeat and rejection, as well as the perception of a lack of social support. These results are consistent with those in literature, with lack of social support or isolation being an especially well-established risk factor [3].

Although the role of impulsivity in suicidal behavior has not been clearly defined yet [50,51], it appears that a considerable proportion of suicide attempts are related to impulsive behavior [52,53]. In our study, we also included personality trait variables. Our results suggest that people at risk at the time of writing their tweet might show less extroversion and less rational personality traits in their posts, which could be associated with greater impulsiveness. Further research would shed more light on this subject.

Although insomnia or sleep problems variables have been identified as risk factors in suicide assessments, our findings suggest that this variable is not identifiable or relevant to social media posts. One study [54] notes that only nightmares are associated with suicidality.

We obtained preliminary data that might help us to predict increased real-time suicide risk. The interpersonal theory of suicide [55] posits that the simultaneous occurrence of 2 psychological states, a perceived burden to others, and a frustrated belonging or social isolation, as well as hopelessness regarding the potential of these states to change, results in the desire for suicide. Our findings are consistent with this theory, showing that sadness, feeling of defeat, or perceived lack of support are related to high-risk tweets. However, according to this theory, the simultaneous presence of suicidal desire and a high tolerance for pain and fear of death would be necessary to produce lethal or near-lethal behavior. The interpersonal theory of suicide also posits that high risk occurs when tolerance for pain and fear increases [49,56]. Our results seem to also suggest that real risk appears when ideation intensity increases and the fear of suicide decreases, making the resulting emotional valence less unpleasant. In other words, the detection of a decrease in fear and, therefore, a less aversive emotional state could predict an increased risk of suicidal behavior at the time on social media. If these variables effectively and consistently prove their ability to predict an increase in risk at the time, we could generate real-time machine learning systems that would detect predictor emotional states, such as the decrease in fear, to prevent potential deaths.

Our study has strengths but also limitations. The cross-sectional design of this study provided limited data about suicidal phenomenon on social networks. In the future, it would be interesting to be able to design a study that screens variation in emotional states and suicide risk over time as this may provide more relevant information on how the risk of suicide varies.

Twitter is considered to be one of the most popular social media platforms, with the greatest immediacy in posting, but because there is a character limit, some variables are barely detectable or measurable. More complex feelings or constructs are difficult to evaluate. Future research could include the evaluation of conversation threads or tweets in their context (for example, by taking into account tweets within the previous 24 hours), which would allow more accurate evaluations of relevant variables in suicide risk assessments.

The interrater reliability was moderate, differences between research and the clinical professional profile of the experts might have had an impact on reliability. In future investigations, reliability will be assessed based on expert profiles, and intensive specific training will be conducted.

One of the strengths of our study is that it incorporates experts in suicide risk assessment. In future research, it would be interesting to introduce validated scales to measure suicidality so that expert risk detection could be effectively validated. Moreover, it would be interesting for future research to use social media users with past suicide attempts to screen and validate our findings.

Our findings—identifying emotional content that might be relevant for real-time suicide prevention—can contribute to the development of new technology-based screening systems; however, more robust research is needed to establish whether social media screening can effectively reduce suicide outcomes and whether there is a way to ethically reach those individuals at risk.

## Abbreviations

API: application programming interface
